# Interplay between programmed death-ligand 1 and non-coding RNAs

**DOI:** 10.3389/fimmu.2022.982902

**Published:** 2022-11-01

**Authors:** Soudeh Ghafouri-Fard, Hamed Shoorei, Bashdar Mahmud Hussen, Yadollah Poornajaf, Mohammad Taheri, Guive Sharifi

**Affiliations:** ^1^ Department of Medical Genetics, School of Medicine, Shahid Beheshti University of Medical Sciences, Tehran, Iran; ^2^ Clinical Research Development Unit of Tabriz Valiasr Hospital, Tabriz University of Medical Sciences, Tabriz, Iran; ^3^ Department of Anatomical Sciences, Faculty of Medicine, Birjand University of Medical Sciences, Birjand, Iran; ^4^ Department of Pharmacognosy, College of Pharmacy, Hawler Medical University, Erbil, Kurdistan, Iraq; ^5^ Center of Research and Strategic Studies, Lebanese French University, Erbil, Kurdistan, Iraq; ^6^ Faculty of Medicine, Birjand University of Medical Sciences, Birjand, Iran; ^7^ Urology and Nephrology Research Center, Shahid Beheshti University of Medical Sciences, Tehran, Iran; ^8^ Institute of Human Genetics, Jena University Hospital, Jena, Germany; ^9^ Skull Base Research Center, Loghman Hakim Hospital, Shahid Beheshti University of Medical Sciences, Tehran, Iran

**Keywords:** PD-L1, lncRNA, miRNA, expression, cancer

## Abstract

Programmed death-ligand 1 (PD-L1) is a transmembrane protein with essential roles in the suppression of adaptive immune responses. As an immune checkpoint molecule, PD-L1 can be exploited by cancer cells to evade the anti-tumor attacks initiated by the immune system. Thus, blockade of the PD1/PD-L1 axis can eliminate the suppressive signals and release the antitumor immune responses. Identification of the underlying mechanisms of modulation of the activity of the PD1/PD-L1 axis would facilitate the design of more efficacious therapeutic options and better assignment of patients for each option. Recent studies have confirmed the interactions between miRNAs/lncRNAs/circ-RNAs and the PD1/PD-L1 axis. In the current review, we give a summary of interactions between these transcripts and PD-L1 in the context of cancer. We also overview the consequences of these interactions in the determination of the response of patients to anti-cancer drugs.

## Introduction

Programmed death-ligand 1 (PD-L1), alternatively named as CD274 or B7-H1 is a transmembrane protein with essential roles in the suppression of adaptive immune responses. The reaction of the adaptive immune system to external or internal danger signals leads to the expansion of antigen-specific CD8+ and/or CD4+ T cell clones (1). However, when PD-L1 binds to the PD-1 checkpoint, an inhibitory signal is transmitted which decreases the proliferation of antigen-specific T-cells in lymph nodes and at the same time reduces apoptosis of regulatory T cells. PD-1/PD-L1 axis mainly acts at the late stage of induction of T-cell immune responses in peripheral tissues (2).

Immune checkpoint pathways are exploited by cancer cells so as to evade the anti-tumor attacks initiated by the immune system (3). Thus, the blockade of immune checkpoints can eliminate the suppressive signals and release the antitumor immune responses. PD-1 and PD-L1 have been used as key drug targets for the development of immune checkpoint blockade treatment modalities (2). Several PD-1/PD-L1 targeted therapies have been approved for the treatment of several types of malignancies (2). At least three human IgG1 antibodies anti-PD-L1 antibodies have been approved for clinical application (4). While atezolizumab and durvalumab have been designed to eliminate FcγR-binding and effector function, avelumab retains the intact function of Fc (5). BMS-936559 is another PD-L1-targeting antibody that is distinctive from the mentioned approved PD-L1 antibodies since it is an IgG4 mAb with S228P mutation (5). In addition, the fusion protein KN035 contains a distinct domain of the humanized anti-PD-L1 antibody and the Fc of an IgG1 (6). Meanwhile, PD-L1-targeting agents can be prescribed in the form of a prodrug. An example is the agent CX-072 which can be activated by a protease (7). Due to incomplete success and drawbacks of using PD-L-targeting drugs, assessment of expression of PD-L1 is regarded as a marker for prediction of response and identification of patients who gain a favorable clinical response from this type of therapy (4).

The expression of genes can be regulated at the transcriptional and posttranscriptional levels by functional RNA molecules known as non-coding RNAs (8, 9). The expression of ncRNAs, which primarily regulate oncogenes and tumor suppressor genes, is altered in several kinds of human malignancies (10, 11). Recent studies have confirmed the interplay between a variety of non-coding RNAs and the PD1/PD-L1 axis. In fact, several microRNAs (miRNAs), long non-coding RNAs (lncRNAs), and circular RNAs (circRNAs) have been shown to modulate the activity of this axis. Identification of molecular mechanisms that modulate the activity of the PD1/PD-L1 axis would facilitate the design of more efficacious therapeutic options and better assignment of patients for each option. In the current review, we give a snapshot of interactions between these transcripts and PD-L1 in the context of cancer. We also overview the consequences of these interactions in the determination of the response of patients to anti-cancer drugs.

## Gastrointestinal cancers

**Figure 1:**
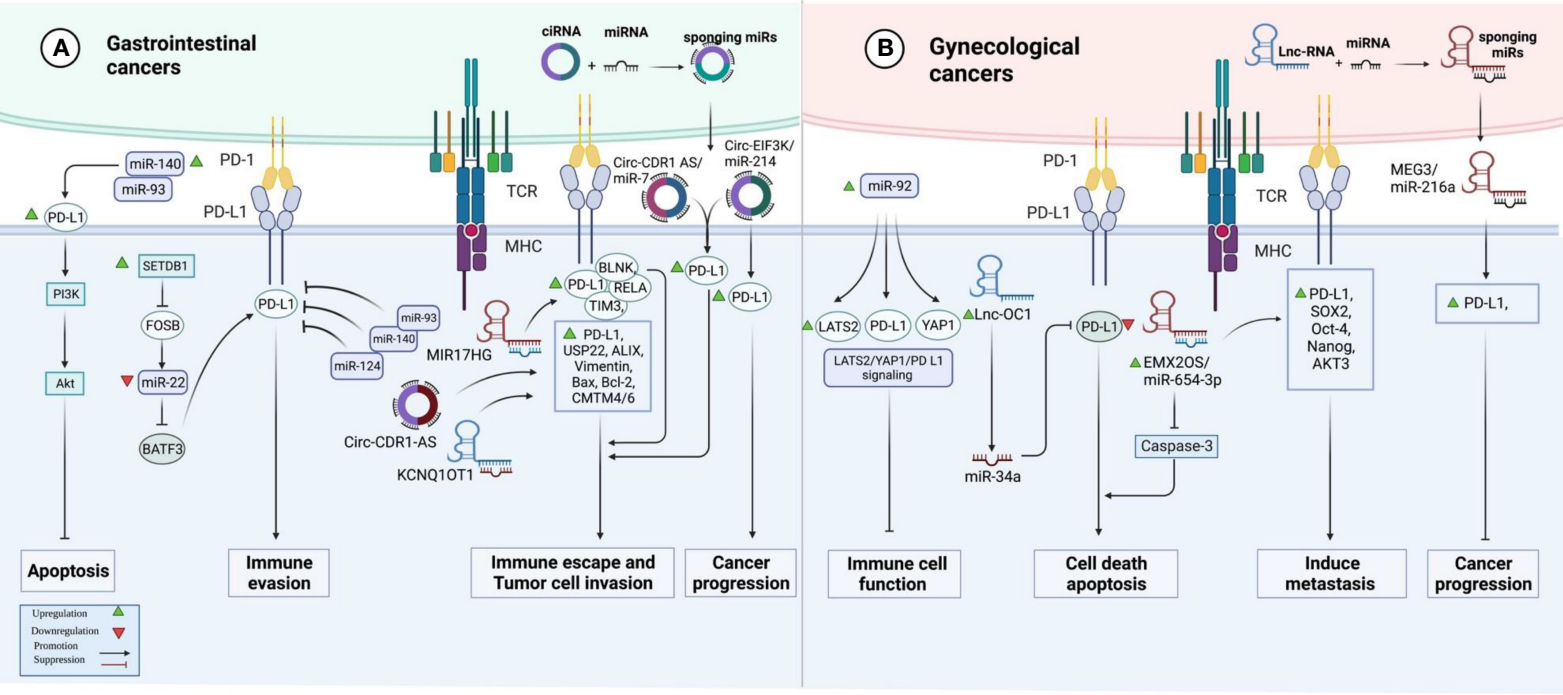
A graphical representation of the ways in which programmed death ligand 1 (PD-L1) and non-coding RNAs interact with one another in gastrointestinal and gynecological cancers.

In colorectal cancer (CRC) samples, over-expression of SETDB1 expression has been associated with the expression of PD-L1. Mechanistically, SETDB1 can down-regulate miR-22 levels by decreasing the expression of FOSB. On the other hand, miR-22 down-regulates PD-L1 levels by targeting BATF3. SETDB1 knockdown has enhanced the cytotoxic effects of T cells on tumor cells by influencing the FOSB/miR-22/BATF3/PD-L1 axis, thus hindering the growth of CRC tumors in mice. Cumulatively, the effects of SETDB1 on the activity BATF3/PD-L1 axis leads to immune evasion of CRC tumors (12). miR-124 is another miRNA with a possible regulatory role on PD-L1. Expression of miR-124 is significantly decreased in CRC, and its under-expression has been correlated with the up-regulation of PD-L1 (13). The luciferase assay has validated PD-L1 targeting by miR-124 (13). miR-124 mimics could significantly reduce the expression of PD-L1 at the transcript, protein, and cell surface levels (13). Moreover, this miRNA could inhibit Tregs in co-culture models by influencing levels of IL-10, IL-2, TNF-α, TGF-β, and IFN-γ. Up-regulation of miR-124 could also decrease the proliferation of CRC cells and induce cell cycle arrest at G1 *via* down-regulating c-Myc. Moreover, this miRNA could induce intrinsic and extrinsic apoptotic pathways, down-regulate CD44 and MMP-9 expression levels, and suppress cell migration and invasion (13). STAT3 signaling has also been suppressed by miR-124 (13). miR‐93‐5p (14) and miR-140-3p (15) are two other miRNAs that can target PD-L1 in CRC cells. Meanwhile, a number of lncRNAs and circRNAs have been found to affect CRC progression by influencing the expression of miRNAs and enhancing the expression of PD-L1. For instance, MIR17HG lncRNA promotes the progression of CRC by influencing the expression of miR-17-5p (16). Besides, KCNQ1OT1 lncRNA released by CRC cells-originated exosomes can mediate immunity through the regulation of PD-L1 ubiquitination *via* miR-30a-5p/USP22 (17). Hsa_circ_0136666 is also involved in Treg-mediated immune escape through modulation of miR-497/PD-L1 axis (**Figure 1A**) (18). Cancer-associated fibroblasts have been shown to secrete circEIF3K in their exosomes to enhance progression of CRC through miR-214/PD-L1 axis (19).

In hepatocellular carcinoma (HCC), suppression of PARP has enhanced the efficacy of immune checkpoint therapy *via* influencing the miR-513/PD-L1 axis. Thus, combined administration of the PARP inhibitor olaparib and anti-PD1 has been suggested as a treatment modality in HCC (20). In this type of cancer, HOXA-AS3 lncRNA has been found to promote proliferation and migratory potential through miR-455-5p/PD-L1 axis (21). Another study in HCC has shown a correlation between expressions of PD-L1 and PD-L2. Moreover, this study has demonstrated the up-regulation of PCED1B-AS1. Expression of PCED1B-AS1 has been positively correlated with expression levels of PD-L1 and PDL-2 while being negatively correlated with hsa-miR-194-5p. Mechanistically, PCED1B-AS1 increases expressions of PD-L1 and PD-L2 through sequestering hsa-miR-194-5p, thus inducing PD-L1/2-mediated immunosuppressive effects on T cells (22). **Table 1** shows the interactions between non-coding RNAs and PD-L1 in gastrointestinal cancers.

**Table 1:** Interactions between non-coding RNAs and PD-L1 in gastrointestinal cancers.

Cancer	miRNA/lncRNA	Sample	Cell Line	PD-L1Expression	Target	Function	Ref
CRC	miR-22 (-)	36 pairs of CRC and paratumoral tissues, BALB/c nude mice	CRL-1831, SW480, FHC, CCL-228, LS174T, CL-188,	Up	BATF3, FOSB	Histone Methyl-transferase SETDB1 *via* BATF3/PD-L1 axis by decreasing miR-22 could enhance immune evasion.	(12)
CRC	miR‐124‐3p (Down-regulated)	20 pairs of CRC and paratumoral tissues	HCT‐116, HT29, SW480, 293T, PBMCs	Negative correlation with miR‐124‐3p	MMP‐9, c-Myc, Bcl-2, Bax, Caspase-3/8/9, STAT3	miR‐124‐3p *via* targeting STAT3 can decrease PD‐L1 expression and block tumorigenesis in CRC cells.	(13)
CRC	miR‐93‐5p (Down-regulated)	125 pairs of CRC and paratumoral tissues	HCT116, SW480, PBMCs	Up	MMP1/2/9, IL‐2/1β/10, IFN‐γ, TGF‐β	miR‐93‐5p *via* targeting PD-L1 could modulate the progression of CRC.	(14)
CRC	miR-140-3p (Down-regulated)	31 pairs of CRC and paratumoral tissues, BALB/c-nude mice	HCT116, SW480, NCM460	Up	PD-L1, PI3K, AKT	miR-140-3p *via* targeting PD-L1 could induce apoptosis and decrease cell growth in CRC.	(15)
CRC	MIR17HG (Up-regulated), miR-17-5p	Cohort study, RELA mice, nude mice	HCT15, HCT116, SW480, SW620, HT29, DLD-1, RKO, LoVo	PD-L1/2, PD-L1 had positive correlation with MIR17HG	BLNK, TIM3, CTLA-4, NF-kB	miR-17-5p is transcribed from MIR17HG and reduces expression of the tumor suppressor B-cell linker. MIR17HG can also upregulate the expression of PD-L1.	(16)
CRC	KCNQ1OT1 (Up-regulated), miR-30a-5p	20 pairs of CRC and paratumoral tissues, BALB/c nude mice	FHC, 293T, SW480, PBMCs SW1463, HT-29, CT26	–	USP22, ALIX, Vimentin, E/N-cadherin, Bax, Bcl-2	LncRNA KCNQ1OT1 *via* miR-30a-5p/USP22 axis by regulating PD-L1 could promote CRC immune escape.	(17)
CRC	Hsa_circ_0136666 (Up-regulated), miR-497	nude mice	HCT116, SW480, SW620, HT29, HCT8, FHC	Up	IL-2/10/1β, TNF-α, TGF-β, AKT, mTOR, ERK1/2, PTEN	Hsa_circ_0136666 *via* targeting miR-497/PD-L1 axis could enhance Treg-mediated immune escape of CRC.	(18)
CRC	Circ-EIF3K (-), miR-214	TCGA database, NOD-SCID mice	HCT116, SW620, FHC, 293T, HDLEC	Up	–	Exosomal circ-EIF3K from cancer-associated fibroblast *via* modulating the miR-214/PD-L1 axis could enhance CRC progression.	(19)
CRC	Circ-CDR1-AS (-), miR-7	BALB/c nude mice	293T, SW620, Caco2, HUVECs	Positive correlation with Circ-CDR1-AS	CMTM4/6	Overexpression of circ-CDR1-AS *via* enhancing cell surface PD-L1 levels could increase the immune escape of CRC cells.	(23)
HCC	miR-513 (-)	C57BL/6 mice	Hep-3b, YY-8103	–	PARP2	Habitation of PARP *via* miR-513/PD-L1 axis in HCC could potentiate immune checkpoint therapy.	(20)
HCC	HOXA-AS3 (Up-regulated), miR-455-5p	TCGA database	Hep3B, SNU-387, Li-7, HuH-7, L-02, 293T	Up	–	Overexpression of lncRNA HOXA-AS3 10 by targeting the miR-455-5p/PD-L1 axis could contribute to cell invasion.	(21)
HCC	PCED1B-AS1 (Up-regulated), hsa-miR-194-5p (Down-regulated)	45 pairs of HCC and paratumoral, nude mice	Huh-7, HepG2, 293T	PD-L1/2, Positive correlation with PCED1B-AS1	LRP6, AKT, STAT3, NoTCH-1	LncRNA PCED1B-AS1 *via* sponging miR-19 by promoting PD-L1 and PD-L2 function could induce immunosuppression in HCC.	(22)
HCC	miR-195 (-)	30 pairs of HCC and paratumoral tissues, wild-type C57BL/6 mice	Hepa1-6, Huh-7, Hep3B, HepG2	–	CHK1, IRF-1, PARP, STAT3	IRF-1 *via* targeting miR-195 by modulating PD-L1 could increase apoptosis of HCC cells.	(24)
HCC	miR-675-5p (-)	152 HCC tissue samples, BALB/C nude mice	SMMC-7721, HepG2. PBMCs	–	HK2, HLA-ABC, EGFR, MAPK	EGFR-P38 MAPK axis *via* miR-675-5p and HK2 by decreasing HLA-ABC could enhance PD-L1 in HCC cells.	(25)
HCC	LINC00657 (Up-regulated), miR-424	60 pairs of HCC and paratumoral tissues	HepG2, Huh7, SMMC-7721, HCCLM3, L02	Up		Knockdown of lncRNA LINC00657 *via* targeting miR-424 by regulating PD-L1 could attenuate HCC cell progression.	(26)
HCC	hsa_circ_0003288 (Up-regulated), miR-145	40 pairs of HCC and paratumoral tissues, BALB/c nude mice	HepG2, Huh7, SMMC-7721, Bel-7402, L02	Up	E/N-cadherin, AKT	hsa_circ_0003288 could induce EMT through modulating the miR-145/PD-L1 axis in HCC cells.	(27)
EAC	miR-145-5p (Up-regulated)	30 pairs of EAC and paratumoral tissues, BALB/c mice	OE33, FLO-1, HET-1A, PBLs	–	SPOP, c-Myb, IFN-γ, IL-2/4/10, MMP-3/9, E/N-cadherin	c-Myb *via* targeting miR-145-5p/SPOP/PD-L1 axis could facilitate immune escape.	(28)
GC	miR-1290 (-)	81 pairs of GC and paratumoral tissues, C3H mice	GEC-1, MGC-803, BGC-823, MFC, 293T	–	Grhl2, ZEB1, TSG101, IL-2, IFN-γ	Tumor-derived extracellular vesicles containing miR-1290 *via* Grhl2/ZEB1/PD-L1 axis could promote the immune escape of cancer cells.	(29)
GC	miR-16-5p (-)	68 pairs of GC and paratumoral tissues, BALB/c mice, NOD/SCID mice	AGS, NCI-N87, 293T, PBMCs	–	iNOS, HSP70, IL-2, TNF-α, INF-γ	miR-16-5p *via* activation of T cell immune response by regulation PD-L1 could inhibit GC progression.	(30)
GC	miR-105-5p (-)	368 GC tissue samples	AGS, NCI–N87, SNU-719, SNU-216, MKN-74, 293T, PBMCs	–	F522, IFN-γ, IL-2	DNA methylation *via* controlling miR-105-5p could decrease PD-L1 expression and increase immunogenicity in GC cells.	(31)
GC	miR-15a/16 (Down-regulated)	6 pairs of GC and paratumoral tissues, BALB/c mice	SGC7901, 293T	Up	TSG101, ALIX	miR-15a/16 *via* modulating PD-L1 could decrease the immune escape of GC cells.	(32)
GC	miR-502-5p (Down-regulated)	25 pairs of GC and paratumoral tissues, nude mice	SGC-7901, BGC823, MGC803, GES-1,	Up	STAT3	miR-502-5p by modulating PD-L1 could enhance GC progression and invasion.	(33)
GC	PROX1-AS1 (Up-regulated), miR-877-5p	30 pairs of GC and paratumoral tissues	AGS, MGC-803, SGC-7901, SNU-1, GES-1	Negative correlation with miR-877-5p	Cyclin-D1, p21, Bax, Bcl-2, Caspase-3/9, Cox-2, MMP-2/9	LncRNA PROX1-AS1 *via* targeting miR-877-5p/PD-L1 axis could accelerate GC progression and invasion.	(34)
GC	HIF1A-AS2 (Up-regulated), miR-429	50 pairs of GC and paratumoral tissues, BALB/c nude mice	SNU-5, HGC-27, MKN45, AGS	Up	–	LncRNA HIF1A-AS2 *via* targeting miR-429/PD-L1 axis could enhance metastasis of GC cells.	(35)
GC	SNHG15 (Up-regulated), miR-141	9 pairs of GC and paratumoral tissues	GES-1, HGC-27, PBMCs	–	–	LncRNA SNHG15 *via* targeting the miR-141/PD-L1 axis could contribute to the immuno-escape of GC cells.	(36)
PC	miR-194-5p (-)	C57BL/6 mice	293T, Panc02, Panc1	Up	N-cadherin, Vimentin, IFN-γ, GZMB	miR-194-5p *via* targeting PD-L1 could regulate the immune escape of PC cells.	(37)
PC	miR-142-5p (-)	C57BL/6 mice	Panc02, 293T, PBMCs	PD-1/PD-L1 (-)	TNF-α, IFN-γ, IL-10	miR-142-5p by blocking the PD-L1/PD-1 axis could enhance anti-tumor immune responses.	(38)
PC	PSMB8-AS1 (Up-regulated), miR-382-3p	90 pairs of PC and paratumoral tissues, BALB/c nude mice	PANC-1, CFPAC, MIA-paca2, AsPC-1, Capan-2, BXPC-3	Up (in the PSMB8-AS1 overexpressed PC cells)	Cyclin-D1, CDK4/6, E-cadherin, Vimentin, STAT1	LncRNA PSMB8-AS1 *via* modulating the miR-382-3p/STAT1/PD-L1 axis could contribute to the PC cells’ progression.	(39)
PC	LINC00473 (Up-regulated), miR-195‐5p	134 PC tissues, 20 normal samples	SW‐1990, 293T, Panc‐1, BxPC‐3, AsPC‐1, CAPAN‐2, H6C7, PBMCs,	Up	Bcl‐2, Bax, IFN‐γ, IL‐4, MMP-2/9	LncRNA LINC00473 *via* modulating PD-L1 by sponging miR‐195‐5p could drive the progression of PC cells.	(40)

## Lung cancer

**Figure 2:**
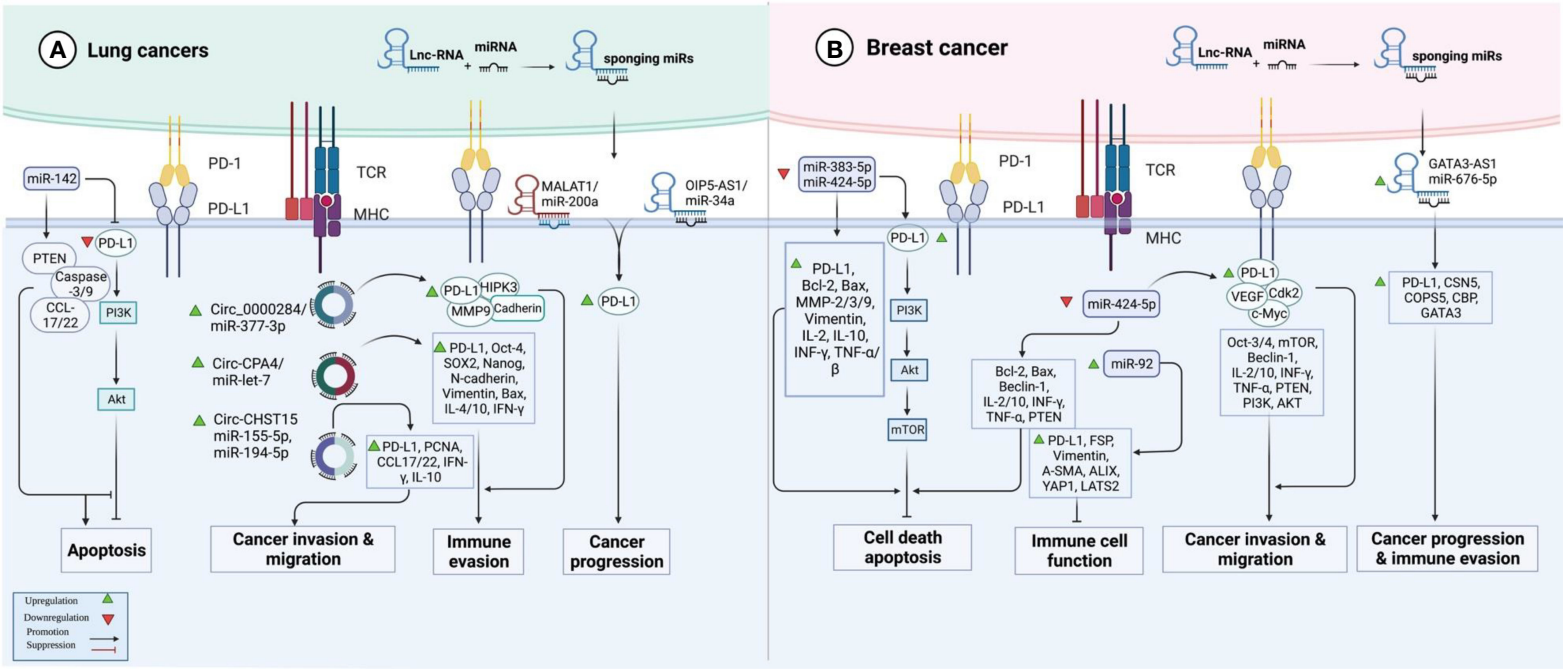
An illustration in graphical form of the methods *via* which the proteins are known as programmed death ligand 1 (PD-L1) and non-coding RNAs such as miRNAs, lnc-RNAs, and circ-RNAs interact with one another in lung and breast cancer.

In lung cancer samples, expression of PD-L1 expression has been correlated with the T stage. Treatment with PD-L1 inhibitor has decreased the expression of PD-L1 and diminished T stage in patients suffering from PD-L1-positive lung cancer. In this group of patients, over-expression of PD-L1 or decreased serum exosomal level of miR-16-5p level has been correlated with longer survival upon treatment with a PD-L1 inhibitor. Moreover, this kind of treatment reduced the quantity of exosomes in the sera of PD-L1-positive patients and enhanced serum exosomal levels of miR-16-5p. High exosomal levels of miR-16-5p could depress cell proliferation and migration in cell cultures, and induce apoptosis, particularly in cells treated with a PD-L1 inhibitor. Cumulatively, the miR-16-5p content of serum exosomes possibly inhibits tumor growth and can be used as a marker for PD-L1 inhibitor therapy (41). miR‐155‐5p (42) and miR-326 (43) are two other miRNAs that suppress PD‐L1 expression and attenuate immune escape in lung cancer by targeting PD-L1. In addition, miR-138-5p has been found to affect the activity of the PD-1/PD-L1 axis, inhibit tumor growth and activate the immune system in this type of cancer (44). A number of oncogenic lncRNAs and circRNAs, namely OIP5-AS1, MALAT1, Circ_0000284, Circ-CPA4 and Circ-CHST15 have been shown to act as molecular sponges for PD-L1-targeting miRNAs, thus incresing levels of PD-L1 (**Figure 2A**) (**Table 2**).

**Table 2:** Interaction between non-coding RNAs and PD-L1 in lung cancer.

miRNA/lncRNA	Sample	Cell Line	PD-L1 Expression	Target	Function	Ref
miR-16-5p (-)	60 LUAD patients and 20 healthy controls, BALB/C nude mice	BEAS-2B, A549, PC9, HCC827	–	Calnexin, TSG101	Serum exosomal miR-16-5p by regulating PD-L1 could inhibit lung cancer progression.	(41)
miR-155-5p (Up)	9 pairs of LUAD and paratumoral tissues	A549, H1650	–	–	miR-155-5p *via* suppressing PD-L1 expression could modulate the immune response in LUAD.	(42)
miR-326 (Down-regulated)	50 LUAD tissue samples, BALB/c mice	A549, H1975, H1734, Calu-3, BEAS-2B, 293T, PBMCs	Negative correlation with miR-326	B7-H3, ICOSLG, TGF-β, IL-2/10/1β, IFN-γ, TNF-α,	miR-326 by modulating PD-L1 and B7-H3 could attenuate immune escape and prevent metastasis in LUAD cells.	(43)
miR-138-5p (-)	5 NSCLC tumor tissue samples, C57BL/6 mice, nude mice	A549, 3LL, 293T	PD-L1/PD-1 (-)	Cyclin-D3, MCM2	miR-138-5p by targeting PD-1/PD-L1 could inhibit tumor growth and activate the immune system.	(44)
miR-142-5p (Up-regulated)	20 NSCLC tissue samples and 20 normal tissue samples, serum samples	A549, 293T, PBMCs	Down	IFN-γ, Caspase-3/9, CCL-17/22, PTEN, PI3K, AKT	miR-142-5p *via* modulating the PTEN pathway by targeting PD-L1 expression could regulate CD4+ T cells in human NSCLC.	(45)
OIP5-AS1 (Up-regulated), miR-34a	68 NSCLC patients	H522, H22, H23	–	–	LncRNA OIP5-AS1 *via* binding to miR-34a could upregulate oncogenic PD-L1 in NSCLC.	(46)
MALAT1 (-), miR-200a-3p	113 NSCLC tissue samples	A549, CAL-12T	Positive correlation with MALAT1	–	LncRNA MALAT1 *via* modulating miR-200a-3p/PD-L1 axis could contribute to NSCLC progression.	(47)
Circ_0000284 (Up-regulated), miR-377-3p	60 pairs of NSCLC and paratumoral tissues, BALB/c nude mice	MRC-5, A549, H82	–	E-cadherin, Fibronectin, MMP9, HIPK3	Circ_0000284 *via* targeting miR-377-3p-mediated PD-L1 surge plays an oncogenic role in NSCLC.	(48)
Circ-CPA4 (Up-regulated), miR-let-7	50 pairs of NSCLC and paratumoral tissues, nude mice	A549, H1299, SK-MES-1, Calu-3, HBE, PBMCs	Up	Oct-4, SOX2, Nanog, N-cadherin, Vimentin, Bax, IL-4/10, IFN-γ	Circ-CPA4 *via* targeting the miR-let-7/PD-L1 axis could regulate immune evasion.	(49)
Circ-CHST15 (Up-regulated), miR-155-5p, miR-194-5p	90 pairs of LC and paratumoral tissues, BALB/c mice	16HBE, H1299, H23, H1359, H1435, A549, H358, PC-9, BNCC341852, CMT-167	Positive correlation with Circ-CHST15	PCNA, CCL17/22, IFN-γ, IL-10	Circ-CHST15 *via* sponging miR-155-5p and miR-194-5p could enhance the invasion and migration of LC cells mediated by PD-L1.	(50)

## Gynecological cancers

The impact of non-coding RNAs on the expression of PD-L1 has been assessed in ovarian cancer (OC), cervical cancer (CC), and endometrial cancer (EC). In OC, miR-92 has been reported to block the functions of immune cells by modulating the expression of PD-L1 *via* the LATS2/YAP1 axis (51). Moreover, the sponging effect of EMX2OS on miR-654-3p has been shown to result in the induction of cell proliferation, invasive properties, and sphere formation in OC through regulation of the AKT3/PD-L1 axis (52). In addition, the Lnc-OC1/miR-34a axis has a crucial role in the pathogenesis of EC through the modulation of PD-L1 (53). Conversely, another study has shown that PD-L1 has a tumor suppressor role in aggressive EC and its expression is influenced by MEG3/miR-216a axis (**Figure 1B**) (54). **Table 3** shows the interactions between non-coding RNAs and PD-L1 in gynecological cancers.

**Table 3:** Interaction between non-coding RNAs and PD-L1 in gynecological cancers.

Cancer	miRNA/lncRNA	Sample	Cell Line	PD-L1 Expression	Target	Function	Ref
OC	miR-92 (Up-regulated)	40 OC tissue samples	SKOV3	–	LATS2, YAP1	miR-92 *via* LATS2/YAP1/PD-L1 signaling overexpression could suppress immune cell function in OC cells.	(51)
OC	EMX2OS (Up-regulated), miR-654-3p	50 pairs of OC and paratumoral tissues, BALB/c nude mice	SKOV-3, ES-2, OVCAR3, A2780, CAOV3, IOSE-80	Up	SOX2, AKT3, Oct-4, Nanog, Caspase-3	LncRNA EMX2OS through modulating miR-654-3p/AKT3/PD-L1 axis could induce metastasis of OC cells.	(52)
EC	Lnc-OC1 (Up-regulated), miR-34a	28 pairs of EC and paratumoral tissues	HESCs, Ishikawa	–	–	Lnc-OC1 by targeting miR-34a and suppressing PD-L1 could induce cell apoptosis in EC.	(53)
EC	MEG3 (Down-regulated), miR-216a	65 EC tissue samples and 18 normal endometrium samples	HEC-50, HeLa, HOUA-I, HEC-1, EM, SPAC-1-L	Down	Caspase-3/7, MCL-1, ZO-1, E-cadherin, EMT, Vimentin, Snail	LncRNA MEG3 *via* repressing miR-216a and by increasing PD-L1 expression could inhibit cell invasion.	(54)

## Breast cancer

At least four studies have assessed interactions between non-coding RNAs and PD-L1 in the context of breast cancer (BCa) (**Table 4**). Two down-regulated miRNAs in BCa, namely miR-383-5p (55) and miR-424-5p (56) have been reported to affect the expression of PD-L1 (**Figure 2B**). Moreover, exosomes secreted by BCa-associated fibroblasts have been shown to assist in the suppression of immune cell function through the transfer of PD-L1 inhibiting miRNA miR-92 (57). Finally, expression of GATA3-AS1 has been found to be markedly increased in triple-negative BCa tissues and cells parallel with a reduction in the proportion of CD8+ T cells. GATA3-AS1 silencing has suppressed the growth of tumor cells and decreased the half-life of the PD-L1 protein. GATA3-AS1 has been shown to induce PD-L1 deubiquitination *via* miR-676-3p/COPS5 axis. Thus, this lncRNA contributes to the progression of triple-negative BCa and immune evasion *via* enhancing the stability of PD-L1 protein and inducing GATA3 degradation (58).

**Table 4:** Interaction between non-coding RNAs and PD-L1 in breast cancer.

miRNA/lncRNA	Sample	Cell Line	PD-L1Expression	Target	Function	Ref
miR-383-5p (Down-regulated)	24 pairs of BCa and paratumoral tissues	MDA-MB-231, PBMCs, MDA-MB-468, MCF-7, SK-BR-3	Up	Caspase-3/9, Bcl-2, Bax, MMP-2/3/9, Vimentin, IL-2, IL-10, INF-γ, TNF-α/β, AKT, PI3K, mTOR	miR-383-5p *via* inhibition of PD-L1 can induce apoptosis and decrease metastasis of BCa cells.	(55)
miR-424-5p (Down-regulated)	35 pairs of BCa and paratumoral tissues	MDA-MB-231, PBMCs, MDA-MB-468, SKBR-3, MCF-7	Up	VEGF, c-Myc, Caspase-3, Bcl-2, Bax, p53, p27, Beclin-1, IL-2/10, INF-γ, PTEN, PI3K, AKT	miR-424-5p *via* targeting PD-L1 could decrease the progression of BCa cells.	(56)
miR-92 (Up-regulated)	34 BCa tissues and 34 normal tissues, BALB/c mice	MA-782, MCF7, NK-92	Up (in cancer cells treated with CAF-derived exosomes)	Vimentin, A-SMA, ALIX, YAP1, LATS2	CAFs *via* targeting the miR-92/PD-L1 axis could suppress immune cell function in BCa cells.	(57)
GATA3-AS1 (Up-regulated), miR-676-5p	68 pairs of TNBC and paratumoral tissues	MDA-MB-468, MDA-MB-436, MDA-MB-231, HCC1937, MCF-10A	–	CSN5, COPS5, CBP, GATA3	GATA3-AS1 *via* stabilizing PD-L1 and degrading GATA3 contributes to TNBC progression and immune evasion.	(58)

## Renal cancer

miR-497-5p is a putative tumor suppressor miRNA in renal cancer whose down-regulation leads to the over-expression of PD-L1 in this type of cancer (59). Moreover, urinary extracellular vesicles (EVs) have been found to contain miR-224-5p. Expression of miR-224-5p has been higher in urinary EVs of patients with renal cell carcinoma compared to healthy controls. Over-expressed miR-224-5p could inhibit proliferation and induce cell cycle arrest by targeting CCND1. Besides, miR-224-5p has a role in the induction of invasive and metastatic properties of renal carcinoma cells. Remarkably, miR-224-5p has a role in the enhancement of PD-L1 stability through the suppression of CCND1 (60). Finally, SNHG1 has been found to regulate immune escape in this type of cancer through targeting miR‐129‐3p and subsequently activating STAT3 and PD‐L1 (61). **Table 5** shows the interaction between non-coding RNAs and PD-L1 in renal cancer.

**Table 5:** Interaction between non-coding RNAs and PD-L1 in renal cancer.

miRNA/lncRNA	Sample	Cell Line	PD-L1 Expression	Target	Function	Ref
miR-497-5p (Down-regulated)	30 pairs of ccRCC and paratumoral tissues, TCGA-KIRC databases	Caki-2, ACHN	Up	–	miR-497-5p suppressing could enhance PD-L1 expression in ccRCC.	(59)
miR-1-3p, miR-150-5p, miR-224-5p (Up-regulated)	35 pairs of RCC and paratumoral tissues, 6 RCC urine samples, and 6 control urine samples	293T, 769-P, 786-O, Caki-1, OS-RC-2, ACHN, 293T, PBMCs	–	Cyclin-D1, GRP94, ALIX, TSG101, SPOP	miR-224-5p containing in urinary extracellular vesicle expression by suppressing Cyclin-D1 could regulate PD-L1 in RCC cells.	(60)
SNHG1 (Up-regulated), miR‐129‐3p	20 pairs of RCC and paratumoral tissues, nude mice	ACHN, A498, 786‐O, Caki‐1, PBMCs	Down (after SNHG1 knockdown)	IL-2, TNF-α, IFN-γ, STAT3	LncRNA SNHG1 *via* targeting miR‐129‐3p by activation STAT3 and PD‐L1 could modulate the immune escape of RCC cells.	(61)

## Other cancers

The interaction between non-coding RNAs and PD-L1 has also been investigated in laryngeal cancer, anaplastic thyroid carcinoma, osteosarcoma, and diffuse large B-cell lymphoma (DLBCL) (**Table 6**). In laryngeal cancer, miR-217 by repressing the AEG-1/PD-L1 axis could inhibit metastasis (62). In thyroid carcinoma, UCA1 has been found to affect the miR-148a/PD L1 axis to attenuate the cytotoxic effects of CD8+T cells (63). In osteosarcoma, miR-200a through promoting PTEN-mediated PD-L1 up-regulation could increase immunosuppression (64). Moreover, LINC00657 *via* targeting miR‐106a could promote metastasis through the activation of PD‐L1 (65). Finally, MALAT1 by sponging miR-195 could enhance tumorigenesis and immune escape of DLBCL (67).

**Table 6:** Interaction between non-coding RNAs and other cancers.

Cancer	miRNA/lncRNA	Sample	Cell Line	PD-L1Expression	Other Targets	Function	Ref
Laryngeal Cancer	miR-217 (Down-regulated)	29 pairs of LC and paratumoral tissues, BALB/C nude mice	Hep2, HUVEC	–	AEG-1	miR-217 by repressing AEG-1/PD-L1 axis expression could inhibit metastasis.	(62)
Anaplastic Thyroid Carcinoma	UCA1 (Up-regulated), miR-148a	10 pairs of ATC and paratumoral tissues, NOG mice	8505C, Hth74, 293T, PBMCs	–	IFN-γ, TNF-α	LncRNA UCA1 *via* targeting miR-148a/PD-L1 axis could attenuate the killing effect of cytotoxic CD8+T cells.	(63)
Osteosarcoma	miR-200a (-)	32 OS tissue samples, Mice	143B, MG63, HOS, U2OS, K7, K7M2, DUNN, PBMCs	Positive correlation with high levels of miR-20a	PD-L1, PTEN	miR-200a could increase immunosuppression.	(64)
	LINC00657 (-), miR‐106a	–	MG63, U2OS, HDLEC, 293T	Up	–	LINC00657 *via* targeting miR‐106a could promote metastasis.	(65)
Diffuse large B-cell lymphoma (DLBCL)	miR-195 (Down-regulated)	20 pairs of DLBCL and paratumoral tissues	OCI-Ly-10, 293T, PBMCs	Up	IFN-γ, TNF-α, IL-10	Overexpression of miR-195 by targeting PD-L1 could attenuate the immune escape of DLBCL.	(66)
	MALAT1 (-), miR-195	37 DLBCL patients	OCI-Ly10, PBMCs	Positive correlation with MALAT1	Slug, E/N-cadherin, Vimentin, ERk1/2	LncRNA MALAT1 by sponging miR-195 could enhance tumorigenesis and immune escape of DLBCL.	(67)

## Interactions between non-coding RNAs and PD-L1 in response to chemotherapeutic agents

The interaction between non-coding RNAs and PD-L1 can also affect the response of cancer cells to anti-cancer modalities. Moreover, anti-cancer drugs can affect these interactions (**Table 7**). Cisplatin is the mostly assessed drug in this regard. Cisplatin *via* modulation of miR-181a expression could negatively regulate PD-L1 expression in NSCLC (68). Meanwhile, miR-3127-5p *via* regulating STAT3 could up-regulate PD-L1-associated chemoresistance in NSCLC cells (69). Besides, miR-526b-3p through inhibiting STAT3-promoted PD-L1 expression could decrease cisplatin resistance and metastasis (70). miR 576 3p by affecting PD L1 and cyclin D1 expression could enhance the cisplatin sensitivity of OC cells (74).

**Table 7:** Chemotherapeutic agents and PD-L1.

Type of Diseases	Drug	Non-coding RNAs	Sample	Cell line	PD-L1 Expression	Other Targets	Function	Ref
NSCLC	Cisplatin	miR-181a (-)	C57BL6/J mice	A549R, H69R (A549, H69, LL/2); treated with 10 μg/ml cisplatin for 24 h	Up (in CDDP-resistant)	ATM, c-FOS	Cisplatin *via* affecting miR-181a expression could negatively regulate PD-L1 expression in NSCLC.	(68)
NSCLC	Cisplatin	miR-3127-5p (-)	64 pairs of NSCLC and paratumoral tissues	A549, NCI-H1299, A549/DDP, 293T, PBMCs	Up	LC3, P62, STAT3	miR-3127-5p *via* regulating STAT3 could up-regulate PD-L1-associated chemoresistance in NSCLC cells.	(69)
NSCLC	Cisplatin	miR-526b-3p (-)	100 NSCLC patients, BALB/c nude mice	BEAS-2B, H1975, A549, PC-9	PD-L1,	MDR1, c-Myc, STAT3	miR-526b-3p through suppression of STAT3-promoted PD-L1 could decrease cisplatin resistance and metastasis.	(70)
NSCLC	Thalidomide	FGD5-AS1 (Up-regulated), miR-454-3p	45 pairs of NSCLC and paratumoral tissues, BALB/c nude mice; treated with 200 mg/kg Thalidomide twice a week for 3 weeks	A549, SPC-A1, H1299, PC-9, H226, 16HBE, 293T, HUVECs; treated with 100 µM thalidomide for 24 h	Up	PD-1, ZEB1, VEGFA, E/N-cadherin	Thalidomide affects FGD5-AS1/miR-454-3p/ZEB1 axis and expressions of VEGFA and PD-1/PD-L1.	(71)
NSCLC	Nobiletin	miR-197 (-)	–	A549, H292, H460; treated with 200 µM Nobiletin for 48 h	PD-L1,	p53, MDM2, STAT3, EGFR, JAK2	Nobiletin by regulating STAT3-mediated PD-L1 expression and *via* p53-independent PD-L1 downregulation could inhibit tumor progression.	(72)
LUAD	Cisplatin	FGD5-AS1 (-), miR-142-5p	46 LUAD tissue samples	A549/DDP, HCC827/DDP, A549, HCC827	Up (in DDP-resistant cells)	–	FGD5-AS1 *via* targeting miR-142-5p/PD-L1 axis could enhance cisplatin resistance of LUAD cells.	(73)
OC	Cisplatin	miR-576-3p (down)	BALB/c nude mice; treated with 10 mg/kg cisplatin, IP, twice a week for 3 weeks	SKOV3/DDP and A2780/DD, 293T, (SKOV3, A2780); treated with 1, 2, 4, 8 µM cisplatin for 48	Up (in DDP-resistant cells)	Cyclin-D1, MDR1, PARP, Caspase-3	miR-576-3p by affecting PD-L1 and cyclin D1 expression could enhance the cisplatin sensitivity of OC cells.	(74)
OC	Cisplatin	miR-145 (Down-regulated)	73 OC tissue samples	A2780, PBMCs, (A2780/DDP); pretreated with 100 μg/ml cisplatin for 24 h	Up (in treated cells with DDP)	c-Myc	Cisplatin-mediated miR-145 downregulation *via* targeting c-Myc by modulating PD-L1 could contribute to cisplatin resistance in OC cells.	(75)
Epithelial Ovarian Cancer	Olaparib, Radiation	miR-200c-3p (-)	5 EOC tissue samples	SKOV3; treated with 1.5 and 5 µM Olaparib after 48 h post-Olaparib treatment, irradiated 4 Gy	PD-L1	c-Myc, β-catenin	miR-200c-3p by combinatorial therapies could contrast the induction of PD-L1 and *via* downregulating the c-Myc/β-catenin could decrease cell proliferation.	(76)
CRC	Polydatin	miR-382 (-)	C57BL/6 mice	293T, (Caco-2, HCT 116); treated with 50, 100, 150 µM Polydatin	Down	PCNA, Caspase-3	Polydatin could induce an antitumor effect by targeting the miR-382/PD-L1 axis in CRC cells.	(77)
Esophageal Cancer	Sevoflurane	Circ-VIM (Up-regulated), miR-124	20 pairs of EC and paratumoral tissues, BALB/C nude mice; treated with 40 mg/kg Sevoflurane, IV, for every 2 days	PBMCs, 293T, HET-1A, TE-10, TE-11, (KYSE-150, Eca-109); treated with 1% to 4% sevoflurane for 6 h	Up	Slug, Fibronectin, E/N-cadherin, Ras, ERK1/2	Circ-VIM silencing has a synergic effect with sevoflurane by regulating the miR-124/PD-L1 axis.	(78)
GC	Cisplatin	NUTM2A-AS1 (Up-regulated), miR-376a	NOD-SCID mice	GES-1, 293 T, HDLEC, (HGC-27, SNU-1); treated with 1 µmol/L cisplatin for 0, 24, 48, 72 h	–	TET1, HIF-1A	LncRNA NUTM2A-AS1 *via* targeting miR-376a by regulating TET1 and HIF-1A could enhance GC tumorigenesis and drug resistance.	(79)
GC	TRAIL	miR-429 (-)	–	MKN45, BGC823, SGC7901, HGC27; treated with 100 ng/ml TRAIL for 6 days	PD-L1	AKT, Caspase-3, EGFR, mTOR	miR-429-mediated regulation of PD-L1 affects the sensitivity of GC cells to TRAIL.	(80)
HCC	Sorafenib (SR)	miR-1 (Up)	SPF BALB/c nude mice	Hep3B, HepG2, 293T, HepG2/SR and Hep3B/SR	Up (in cells treated with SR)	NRF-2, P-gp, MRP1,	NRF-2/miR-1/regulatory axis *via* targeting PD-L1 contributes to the development and maintenance of drug resistance.	(81)
HCC	SR	KCNQ1OT1 (Up), miR-506	SR-sensitive (n=25) and SR resistant (n=38) HCC tissue samples	SK-HEP-1, Huh-7, SK-HEP-1/SR, Huh-7/SR; treated with 1-3 µM Sorafenib for 48 h	Positive correlation with KCNQ1OT1	TNF-α, IFN-γ, IL2/10, TGF-β	LncRNA KCNQ1OT1 *via* sponging miR-506 could contribute to SR resistance and PD-L1-mediated immune escape in HCC cells.	(82)
BCa	Oleuropin	miR-194 (-)	21 pairs of BC and paratumoral tissues	MDA-MB-231; treated with 200 μM Oleuropin for 72 h	Up	XIST	Oleuropin *via* modulating the miR-194/XIST/PD-L1 axis could repress BCa progression.	(83)
BCa	Adriamycin (ADR)	miR-3609 (Down-regulated)	47 BCa tissue samples and 19 paratumoral tissues	293T, HBL-100, MCF-7, MDA-MB-231, MDA-MB-468, (MCF-7/ADR); treated with 0.001, 0.01, 0.1, 1, 10, 100 μg/mL ADR for 48 h	Up (in treated cells with ADR)	–	miR-3609 by blocking the PD-L1 immune checkpoint could sensitize BCa cells to ADR.	(84)
Glioma	Paclitaxel	miR-34a (Down-regulated)	21 glioma patients	U251, U87-MG, U87/P; treated with 0-200 nM Paclitaxel for 24-72 h	Up	Caspase-3	miR-34a *via* targeting PD-L1 could attenuate glioma cell invasion and chemoresistance.	(85)

Thalidomide has been shown to suppress angiogenic process and immune evasion of NSCLC cells by influencing the expression of VEGFA and PD-1/PD-L1 *via* affecting FGD5-AS1/miR-454–3p/ZEB1 axis (71). Moreover, nobiletin has been shown to regulate PD-L1 expression through the modulation of STAT3, thus inhibiting the progression of NSCLC (72). Meanwhile, miR-34a *via* targeting PD-L1 could attenuate glioma cell invasion and their chemoresistance (85).

## Discussion

PD-L1 has been found to participate in the tumorigenesis process *via* induction of Tregs and inhibition of antitumor immune responses. Besides, several non-coding RNAs can act as regulators of gene expression, particularly at the posttranscriptional level to affect the expression of PD-L1. In fact, a delicate interactive network exists between non-coding RNAs that influence the expression of PD-L1. Several lncRNA/miRNA or circRNA/miRNA modules have been found in this network. MIR17HG/miR-17-5p, KCNQ1OT1/miR-30a-5p, hsa_circ_0136666/miR-497, circ-EIF3K/miR-214, circ-CDR1-AS/miR-7, HOXA-AS3/miR-455-5p, PCED1B-AS1/hsa-miR-194-5p, LINC00657/miR-424, LINC00657/miR-424, hsa_circ_0003288/miR-145, PROX1-AS1/miR-877-5p, HIF1A-AS2/miR-429, SNHG15/miR-141, PSMB8-AS1/miR-382-3p, LINC00473/miR-195‐5p, OIP5-AS1/miR-34a, MALAT1/miR-200a-3p, circ_0000284/miR-377-3p, circ-CPA4/miR-let-7, circ-CHST15/miR-155-5p, circ-CHST15/miR-194-5p, EMX2OS/miR-654-3p, Lnc-OC1/miR-34a, MEG3/miR-216a, GATA3-AS1/miR-676-5p, LINC00657/miR‐106a and MALAT1/miR-195 are examples of these modules.

In addition to the above-mentioned PD-L1-interacting non-coding RNAs, several non-coding RNAs can indirectly affect the activity of the PD-1/PD-L1 pathway (86). For instance, the up-regulated lncRNA in diffuse large B cell lymphoma SNHG14 adsorbs miR-5590-3p to enhance expression levels of ZEB1, thus activating the PD-1/PD-L1 pathway and inducing immune evasion (87).

The interactions between non-coding RNAs and PD-L1 are also involved in the response of patients to several anti-cancer modalities. These interactions can also be affected by the administration of several treatment modalities. Cisplatin, Adriamycin, Paclitaxel, Thalidomide, Nobiletin, Olaparib, Sevoflurane, Sorafenib, and Oleuropin are examples of drugs that can affect/be affected by such interactions.

Most importantly, several PD-L1-related non-coding RNAs have been found to be secreted in exosomes originated from tumor cells’ microenvironment residing cells. This finding not only shows the extensive impacts of these non-coding RNAs in tumor progression or modulation of the tumor microenvironment but also potentiates these transcripts as biomarkers for the early detection of cancer using biofluids.

The promising results of clinical trials of anti-PD-L1 during recent years have encouraged researchers to find better treatment modalities to enhance the clinical response to these modalities. Non-coding RNAs are putative modulators of PD-L1 expression and activity, and thus can be used as therapeutic targets in this regard. Moreover, expression levels of PD-L1-associated non-coding RNAs can be used as predictive markers for response to PD-L1 inhibitors. In fact, the lack of appropriate response to this type of therapy can be attributed to the levels of expressions of PD-L1-associated non-coding RNAs. Thus, modulation of expression of these transcripts using siRNA or antisense oligonucleotides can enhance clinical response to anti-PD-L1 therapy. Application of these methods needs a prior identification of the pattern of expression of PD-L1-associated non-coding RNAs in patients’ samples and the functional network between these transcripts and PD-L1. This can be achieved using high throughput sequencing methods with a system biology approach. Future studies are needed to propose tissue-specific panels of non-coding RNAs for this purpose.

## Data availability statement

The analyzed data sets generated during the study are available from the corresponding author upon reasonable request.

## Author contributions

SG-F wrote the manuscript and revised it. MT and GS supervised and designed the study. HS, YP and BH collected the data and designed the figures and tables. All authors contributed to the article and approved the submitted version.

## Acknowledgments

The authors would like to thank the Clinical Research Development Unit (CRDU) of Loghman Hakim Hospital, Shahid Beheshti University of Medical Sciences, Tehran, Iran for their support, cooperation, and assistance throughout the period of. The authors want to acknowledge the clinical research development unit of Tabriz Valiasr Hospital for their support of this study.

## Conflict of interest

The authors declare that the research was conducted in the absence of any commercial or financial relationships that could be construed as a potential conflict of interest.

## Publisher’s note

All claims expressed in this article are solely those of the authors and do not necessarily represent those of their affiliated organizations, or those of the publisher, the editors and the reviewers. Any product that may be evaluated in this article, or claim that may be made by its manufacturer, is not guaranteed or endorsed by the publisher.

## Abbreviations

CRC, Colorectal Cancer; CC, Cervical cancer; OC, Ovarian Cancer; EC, Endometrial Cancer; HCC, Hepatocellular Carcinoma; EAC, Esophageal Adenocarcinoma; GC, Gastric Cancer; PC, Pancreatic Cancer; LUAD, Lung Adenocarcinoma; NSCLC, Non-Small Cell Lung Cancer; LC, Lung Cancer; PD-L1, Programmed Death-Ligand 1; TNBC, Triple-Negative Breast Cancer; BCa, Breast Cancer; IP, Intraperitoneal; PARP, Polymerase; OIP5-AS1, OPA-Interacting Protein 5 Antisense Transcript 1; TAMs, Tumor-Associated Macrophages; HK2, Hexokinase 2; EGFR, Epidermal Growth Factor Receptor; SPOP, Cullin 3-Speckle Type POZ Protein; IV, Intravenous; FGD5-AS1, FGD5-Antisense 1; Grhl2, Grainy Head-Like 2; HIF1A-AS2, Hypoxia-Inducible Factor-1 Alpha Antisense RNA-2; CHK1, Checkpoint Kinase 1; IRF-1, Interferon Regulatory Factor 1; HUVECs, Human Umbilical Vein Endothelial Cells; CIK, Cytokine-Induced Killer; HUSMC, Human Uterine Smooth Muscle Cells; HDLEC, Human Dermal Lymphatic Endothelial Cells; HESCs, Human Embryonic Stem Cells; PTEN, Chromosome Ten; MMP‐1, Metalloproteinase‐1; NPs, Nanoparticles; CAFs, Cancer-Associated Fibroblasts; NRF-2, Nuclear Factor E2-Related Factor 2; TRAIL, Tumor Necrosis Factor-Related Apoptosis-Inducing Ligand; dMMR, Mismatch Repair Deficiency; BLNK, B-cell linker; PBLs, Peripheral Blood Lymphocytes.
